# Changes in insomnia severity with advanced PAP therapy in patients with posttraumatic stress symptoms and comorbid sleep apnea: a retrospective, nonrandomized controlled study

**DOI:** 10.1186/s40779-019-0204-y

**Published:** 2019-05-09

**Authors:** Barry J. Krakow, Natalia D. McIver, Jessica J. Obando, Victor A. Ulibarri

**Affiliations:** 1 Sleep & Human Health Institute, 6739 Academy Rd NE Ste380, Albuquerque, NM 87109 USA; 2 Maimonides Sleep Arts & Sciences, 6739 Academy Rd NE Ste380, Albuquerque, NM 87109 USA; 3 Los Alamos Medical Center, 3917 W Rd, Los Alamos, NM 87544 USA; 4 Institution: Mozaik Solutions, Solana Beach, CA 92075 USA

**Keywords:** Insomnia, Obstructive sleep apnea, Complex insomnia, CBT-I, Posttraumatic stress symptoms, CPAP, Auto bi-level, Adaptive servo-ventilation

## Abstract

**Background:**

Sleep disorders frequently occur in posttraumatic stress disorder (PTSD) patients. Chronic insomnia is a common feature of and criteria for the diagnosis of PTSD. Another sleep disorder, obstructive sleep apnea (OSA), also occurs frequently in PTSD, and emerging research indicates OSA fuels chronic insomnia. Scant research has investigated the impact of OSA treatment on insomnia outcomes (Insomnia Severity Index, ISI) in trauma survivors.

**Methods:**

OSA patients with moderately severe posttraumatic stress symptoms were studied in a retrospective chart review. Ninety-six patients who failed CPAP therapy due to expiratory pressure intolerance or complex sleep apnea or both underwent manual titration with advanced PAP modes [autobilevel (ABPAP); adaptive servo-ventilation (ASV)], which were subsequently prescribed. PAP use measured by objective data downloads divided the sample into three groups: compliant regular users (C-RU): *n =* 68; subthreshold users (SC-RU): *n =* 12; and noncompliant users (NC-MU): *n =* 16. The average follow-up was 11.89 ± 12.22 months. Baseline and posttreatment ISI scores were analyzed to assess residual insomnia symptoms as well as cure rates.

**Results:**

The C-RU group showed significant improvements in insomnia with very large effects compared to those in the NC-MU reference group (*P = 0.*019). Insomnia severity significantly decreased in all three groups with large effects (C-RU, *P = 0.*001; SC-RU, *P = 0.*027; NC-MU, *P = 0.*007). Hours of weekly PAP use and insomnia severity were inversely correlated (*P = 0.*001, *r* = − 0.321). However, residual insomnia symptoms based on established ISI cut-offs were quite common, even among the C-RU group. Post hoc analysis showed that several categories of sedating medications reported at baseline (hypnotics, anti-epileptic, opiates) as well as actual use of any sedating medication (prescription or nonprescription) were associated with smaller insomnia improvements than those in patients not using any sedating agents.

**Conclusions:**

In a retrospective, nonrandomized analysis of a select sample of sleep clinic patients with OSA and PTSD symptoms, advanced PAP therapy was associated with significant improvement in insomnia severity for both compliant and partial users. However, residual insomnia symptoms persisted, indicating that PAP therapy provides only limited treatment. RCTs are warranted to assess the effect of ABPAP and ASV modes of therapy on adherence and sleep outcomes, and their potential impact on posttraumatic stress symptoms. Treatment arms that combine PAP with CBT-I would be expected to yield the greatest potency.

## Background

Chronic insomnia is an extremely common feature of posttraumatic stress disorder (PTSD), and insomnia research on trauma survivors has elaborated on two salient clinical characteristics: (a) insomnia is the most common presenting complaint expressed by PTSD patients [1]; and (b) insomnia is a significant postdeployment predictor of PTSD [2]. Thus, treatment of insomnia in certain trauma survivors would provide substantial benefits by offering relief for one of their most vexing symptoms and which may potentially improve PTSD outcomes. In fact, several studies have already demonstrated that PTSD patients treated for insomnia report favorable outcomes for insomnia or posttraumatic stress symptoms or both [3–8].

Pharmacotherapy [9] or cognitive-behavioral therapy for insomnia (CBT-I) [5] are conventional though not entirely evidence-based approaches to treat insomnia in PTSD, and both modalities have yielded medium to large effects in various trauma survivor cohorts in a few studies of crime victims [10], disaster survivors [11], and military cohorts [5, 7, 12]. Nonetheless, clear evidence among other insomnia cohorts without PTSD strongly affirms the substantial benefits of CBT-I [13, 14].

In some PTSD cases, insomnia may be related to co-occurring obstructive sleep apnea (OSA) [15], a comorbidity originally termed “complex insomnia” in a sample of crime victims with posttraumatic stress symptoms [16]. OSA is a physiological sleep disorder that manifests at surprisingly high rates in trauma survivors [17, 18] as well as in chronic insomnia patients [16, 19–23]. Furthermore, a growing corpus of research has demonstrated an association between OSA treatments and decreases in insomnia severity [22, 24–28]. Taken together, the question arises whether to expect similar outcomes when OSA is treated in PTSD patients. Specifically, would a trauma survivor with OSA treated with positive airway pressure (PAP) therapy manifest a favorable change in insomnia symptom severity? And, to what extent would residual insomnia symptoms persist?

Although treatment research in the area of insomnia, PTSD, and OSA is sparse, there are some indications that sleep breathing treatments may improve insomnia outcomes [24–26, 28–31]. To our knowledge, the first study to examine a case series of trauma survivors with sleep onset or sleep maintenance insomnia and comorbid sleep apnea demonstrated very high rates of self-reported improvement in sleep (“sleeping better”) with the use of continuous positive airway pressure (CPAP) [32]. A second study in 2004 investigated a group of 17 crime victims with posttraumatic stress symptoms presenting with insomnia; they were followed prospectively through stepwise interventions, starting with CBT-I [10] and concluding with CPAP for OSA, both of which resulted in substantial insomnia severity improvements from the combined therapies with large effects for either CBT-I (*d* = 1.53) or CPAP (*d* = 1.11) [27]. However, in clinical terms, only 8 of 17 participants (47%) reached a nonclinical insomnia level [insomnia severity index (ISI) < 11] at CBT-I follow-up, whereas 15 of 17 (88%) achieved this state at the study endpoint, suggesting a potential for greater therapeutic effects from PAP therapy. More likely, the study supported the necessity for both treatments in patients with this complex insomnia comorbidity. A decade later, Amin et al. [29] studied two small PTSD-OSA groups receiving either exposure therapy and autoadjusting CPAP (APAP) or exposure therapy only; the former group with the combination protocol demonstrated a 36% decrease in ISI while the exposure therapy only group demonstrated no change or worsening (*P =* 0.015). In a recent study in PTSD patients, El-Solh and colleagues demonstrated both CPAP and oral appliance therapy improved insomnia severity measured on the Pittsburgh Sleep Quality Index (PSQI) [33].

One barrier that may impede research on the relationship between insomnia and OSA in trauma survivors is the well-described difficulty with CPAP adaptation in PTSD patients [34, 35]. All PAP attempters, with or without PTSD, encounter many issues when trying PAP, such as nasal irritation, congestion, rhinorrhea, dry mouth/throat, pressure in the ears, aerophagia, claustrophobia, skin or eye irritation, nasal pressure sores, skin creases, mouth breathing, mask pain, and mask leak [36]. In PTSD patients, we have noted a heightened sensitivity to the problem of expiratory pressure intolerance (EPI), necessitating the use of advanced PAP modes such as auto bi-level (ABPAP) or adaptive servo-ventilation (ASV). The development of this technology-driven paradigm began in 2005 after we first observed the phenomenon of objective expiratory intolerance and the failure to resolve it with CPAP, CFlex or other EPR technologies. Since 2008, compared to CPAP, we have noted that ABPAP or ASV devices have a much greater ability to smooth the airflow curve [37] on both inspiration and expiration, the latter among patients meeting complex sleep apnea diagnosis (central apnea index (CAI) > 5; CAI/AHI > 50%). In fact, we recently demonstrated higher than usual adherence rates (58%) in PTSD patients using these advanced PAP modes [38] in contrast to commonly reported lower compliance rates (~ 30%) [39] in this vulnerable population. Therefore, we have speculated that a major advantage of ASV or ABPAP over CPAP is the capacity for advanced modes to prevent or eradicate EPI, a principal cause of CPAP rejection [40, 41].

To provide further evidence on the effects of advanced PAP therapy on insomnia in PTSD patients with comorbid OSA, we conducted a retrospective, nonrandomized controlled study of a consecutive series of trauma survivors. The patients provided objective data downloads and subjective follow-up outcomes. We divided our sample into three groups based on PAP adherence: compliant, subthreshold compliant (see Methods), and a reference group of noncompliant patients. We hypothesized that compliant and subthreshold compliant patients would manifest clinically relevant treatment effects on insomnia severity compared to the effects observed in the noncompliant group, but that residual insomnia symptoms would persist in the majority of patients.

## Methods

### Informed consent

Patients provided consent at intake at Maimonides Sleep Arts & Sciences (MSAS) to use their information anonymously for research purposes. All data were de-identified for this case series. The Los Alamos Medical Center Institutional Review board found the chart review exempt.

### Intake and follow-up measures

MSAS requires patients to complete an online intake assessing sleep symptoms and sleep indices [sleep onset latency (SOL), sleep efficiency (SE), and wake after sleep onset (WASO)] based on the nosology for sleep disorders as defined in the International Classification of Sleep Disorders [42] as well as the validated ISI [43]. The ISI is a 7-item questionnaire (scored on a Likert scale from 0 to 4, max total score 28) assessing insomnia symptom severity and resulting impairment: 0–7 no clinical insomnia, 8–14 mild insomnia, 15–21 moderate insomnia, 22 or above severe insomnia. At various intervals after initiation of PAP therapy, patients return for follow-up based on insurance-driven timetables, patient-driven requests for problem-solving, or through a reminder in our sleep center’s detailed follow-up system. For this study, the most recent follow-up data were tabulated. At intake only, patients complete the PTSD symptom scale (PSS) [44], which comprises 17 questions scored on a Likert scale from 0 to 3 with a total range of 0 to 51. PSS > 21 are consistent with moderately severe posttraumatic stress symptoms among individuals who report a history of traumatic exposure.

### Sample and inclusion criteria

This retrospective chart review included adult patients (> 18 years), presenting to MSAS between December 2009 and March 2017, who met the following criteria: 1) history of traumatic exposure and PSS score ≥ 21; 2) objectively diagnosed OSA [Apnea Hypopnea Index (AHI) > 5] or upper airway resistance syndrome (UARS) [AHI < 5 and Respiratory Disturbance Index (RDI) > 15]; 3) CPAP failure; 4) completion of attended, manual titration (or split-therapy PSG [45]) with advanced PAP; 5) filled prescription for advanced PAP therapy with attempted home use; and 6) completed follow-up appointment to assess outcomes and objective data downloads (ODD).

**Fig. 1:**
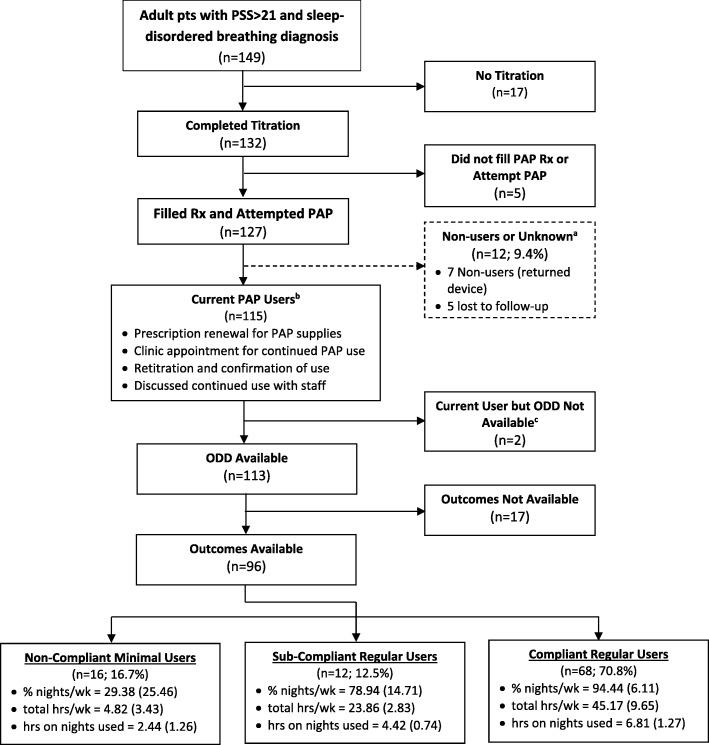
Flowchart showing inclusion and exclusion criteria resulting in the 96 patients comprising our three compliance groups. PSS: PTSD Symptom Scale; PAP: positive airway pressure; ODD: objective data download; ^a^ No evidence available to prove patient is using or attempting to use PAP; ^b^ Current PAP Users based on any one or more of the criteria in the box; ^c^ Met use criteria but no ODD available

Figure 1 describes the selection process for entry into the chart review, which commenced with 149 eligible patients, but 17 did not complete titration PSG, and 5 did not fill a prescription. Thus, 127 attempted PAP of which 115 were current PAP users at the most recent follow-up (see Fig. 1 for use criteria). However, outcomes or ODDs were not available for 19 of the 115 current PAP users, yielding a final sample of 96 patients. There were no systematic differences in sociodemographics, subjective intake sleep indices, or objective sleep breathing indices between our final sample of 96 users with ODD and the 19 patients excluded from analysis due to their lack of data.

### PAP titration protocol in the context of CPAP failure

In a recently published study, we describe our use of advanced PAP therapy devices in PTSD patients who often fail the fixed CPAP mode of treatment primarily due to expiratory pressure intolerance (EPI) or iatrogenic central apneas or both [38]. Many patients fail at initial exposure during the titration itself or even earlier during the desensitization procedure conducted at the presleep portion of overnight polysomnography [46, 47]. In our experience, vulnerable patients (e.g. psychiatric comorbidity) are particularly susceptible to either scenario, and similar side-effects may also unfold during any part of a titration if a patient awakens and is not tolerating CPAP [46, 47].

In our clinical and research experience, described in several papers of psychiatric patients who complain about CPAP [30, 31, 46, 48, 49], we offer a theory linking PAP discomfort and intolerance to the use of fixed pressurized air. The sensation of breathing out against pressurized air yields an uncomfortable, if not intolerable, physiological feeling that the patient may identify (subjective EPI), or the sleep technologist may observe as irregularities on the expiratory limb of the airflow curve (objective EPI) [48]. In actuality, patients may also use stronger subjective terms such as “suffocating” to describe these experiences. Once a pattern of expiratory intolerance emerges, we have observed that psychiatric patients in general and PTSD patients in particular with their tendencies toward anxiety sensitivity [50] are highly susceptible to the development of an attention amplification response [51–53], which we speculate magnifies feelings of discomfort and intolerance not unlike somatosensory amplification observed with conditions such as pain in migraine headache [54], susceptibility to drug side-effects [55], and dyspnea in patients with asthma [56].

Regardless of how the experience of discomfort or intolerance emerges in psychiatric patients, this discomfort or intolerance may deteriorate to panic or frank claustrophobic feelings where patients report they are “drowning in air” [57, 58]. In some situations, we are aware of OSA or UARS patients having been pushed to continue attempting CPAP in a lab environment or at home, which then elicited further adverse emotional responses, including frustration, anger, discouragement, anxiety and fear; less commonly, but not rarely, a patient may report the experience as traumatizing, albeit the sleep literature regrettably has never established a prevalence for this most severe form of CPAP rejection [38, 59]. For these reasons, discretion has guided us to switch individuals to an expiratory relief mode, which may rapidly enhance comfort and evoke a more positive attitude and outcome [46, 47].

Finally, it is worth clarifying that if patients attempt standard PAP modes at home, during presleep desensitization or during titration polysomnography (PSG), then subjective or objective CPAP failure is sufficient to escalate the patient’s care to merit a trial with an advanced PAP mode. However, some insurers or durable medical equipment (DME) companies may mandate home CPAP use as a prerequisite to the declaration of CPAP failure, which we find alarming in the context of patients who report traumatizing experiences with fixed pressure. Finally, in our extensive clinical experience, the manual titration of ABPAP or ASV in the sleep lab has proven consistently superior to home use of these same advanced devices when prescribed at the arbitrary default settings. Unequivocally, the sleep laboratory plays a crucial role in this protocol [60–62].

Accordingly, in the sample for this chart review, all patients had failed CPAP at either our center (*n =* 50) or at a previous sleep center (*n =* 46). As noted above [36], many other factors may interfere with attempts to use PAP. While these other factors were addressed in this sample, patients still reported or demonstrated other chronic issues, notably EPI, persistent central apneas, or the patient proved intolerant to CPAP during either the desensitization or titration PSG at our center. CPAP failure also manifested in current CPAP users as non-adherence or poor outcomes coupled with overtly expressed dissatisfaction about CPAP therapy.

### Compliance metrics & PAP modes

The patients with objective compliance data were divided into three subgroups: compliant regular users (C-RU), patients averaging > 4 h/night on > 70% of nights used, thus meeting Center for Medicare and Medicaid Services (CMS) criteria; sub-compliant regular users (SC-RU), patients using PAP regularly for nightly hours or nights per week approaching but not meeting CMS criteria; and non-compliant minimal users (NC-MU), patients with minimal PAP use and averaging far less than < 2 h/night or 5 nights per week. There were no systematic differences between the three groups in any baseline characteristics including sociodemographic and subjective and objective sleep indices (Table 1).

**Table 1:** Baseline characteristics of total sample and compliance groups: Compliant Regular Users, Sub-Compliant Regular Users, and Non-Compliant Minimal Users^a^

Item	Total sample (*n =* 96)	Compliant regular users (C-RU, *n =* 68)	Sub-compliant regular users (SC-RU, *n =* 12)	Non-compliant minimal users (NC-MU, *n =* 16)	*P*-value^b^
Sociodemographic					
Female [*n*(%)]	49 (51.0)	35 (51.5)	5 (41.7)	9 (56.3)	0.741
Age (year)	49.29 ± 12.96	50.30 ± 13.06	43.88 ± 12.72	49.05 ± 12.47	0.288
BMI (kg/m^2^)	33.95 ± 10.63	34.75 ± 11.14	35.81 ± 11.74	29.14 ± 5.41	0.133
Caucasian [*n*(%)]	55 (57.3)	43 (63.2)	5 (41.7)	7 (43.8)	0.185
Hispanic [*n*(%)]	31 (32.3)	20 (29.4)	5 (41.7)	6 (37.5)	0.625
Some college or less [*n*(%)]	59 (61.5)	40 (58.8)	9 (75.0)	10 (62.5)	0.567
Married/Living with partner [*n*(%)]	50 (52.1)	33 (48.5)	8 (66.7)	9 (56.3)	0.175
Baseline					
PSS	30.67 ± 8.11	29.97 ± 7.64	30.33 ± 6.02	33.88 ± 10.76	0.215
ISI	20.47 ± 5.01	19.96 ± 5.41	20.83 ± 4.06	22.38 ± 3.32	0.222^c^
Sleep indices					
AHI-diagnostic	27.97 ± 27.83	30.34 ± 31.22	16.83 ± 11.69	26.29 ± 18.04	0.294
RDI-diagnostic	51.58 ± 35.47	49.59 ± 38.74	50.95 ± 24.35	60.51 ± 26.97	0.545
Subjective sleep metrics					
SOL (min)	94.72 ± 92.41	87.09 ± 78.75	97.58 ± 89.80	125.00 ± 138.65	0.338
SE (%)	71.9 ± 18.0	72.6 ± 17.7	81.6 ± 23.7	61.9 ± 23.0	0.029^c^
WASO (min)	126.21 ± 113.63	110.71 ± 101.67	104.42 ± 109.77	208.44 ± 136.08	0.006^c^
Medications [*n*(%)]					
Hypnotics	42 (43.8)	26 (38.2)	7 (58.3)	9 (56.3)	0.235
Mood Stabilizers	54 (56.3)	38 (55.9)	6 (50.0)	10 (62.5)	0.799
Opiates	15 (15.6)	9 (13.2)	1 (8.3)	5 (31.3)	0.154
Over the Counter	28 (29.2)	22 (32.3)	1 (8.3)	5 (31.3)	0.236
Seizure Medication	17 (17.7)	11 (16.2)	2 (16.7)	4 (25.0)	0.704

*BMI* Body mass index, *PSS* PTSD Symptom scale, *ISI* Insomnia severity index, *AHI* Apnea hypopnea index, *RDI* Respiratory disturbance index, *SOL* Sleep onset latency, *SE* Sleep efficiency, *WASO* Wake after sleep onset. ^a^Average expressed as: % or mean ± SD; ^b^*P*-value obtained using: 3-way ANOVA for continuous variables, Chi Square for dichotomous variables; ^c^Exploratory analysis for significance and effect size in relevant variable by group comparisons:

i) Finding of significance for ISI: C-RU vs. NC-MU (*P = 0.*91; g = 0.47); SC-RU vs. NC-MU (*P = 0.*597; g = 0.16); C-RU vs. SC-RU (*P = 0.*276; g = 0.41)

ii) Finding of significance for SE: C-RU vs. NC-MU (*P = 0.*043, g = 0.56); SC-RU vs. NC-MU (*P = 0.*036, g = 0.82); C-RU vs. SC-RU (*P = 0.*129, g = 1.48)

iii) Finding of significance for WASO: C-RU vs. NC-MU (*P* = 0.002, g = 0.89); SC-RU vs. NC-MU (*P = 0.*039, g = 1.80); C-RU vs. SC-RU (*P = 0.*846, g = 0.06)

The use of the subthreshold group warrants further comment. Increasing attention has been given to the arbitrary nature of the CMS compliance metrics [63]. Indeed, the American Thoracic Society has noted several studies showing benefits to patients using PAP at insufficient levels not meeting CMS compliance metrics [63–66] and has formally declared, “We consider patients adherent if…they use CPAP for more than 2 h/night and are making progress toward improved daytime sleepiness as measured by the ESS, subjective improvement in quality of life, or improvement of other OSA-associated health impairments (e.g., diabetes, hypertension” [63]. Aligned with these views, a dose-response relationship has been offered as another way to appreciate this continuum between noncompliant and compliant use of PAP therapy [67].

### Data analysis

Descriptive baseline variables were analyzed for the 3 groups with ANOVA. Repeated measures ANOVA compared pre- and post-treatment outcome measures for ISI continuous variables within and between subjects, and effect sizes were calculated with Hedges’ g due to small or unequal sample sizes. Contingency coefficients compared baseline differences among dichotomous variables. ANOVA was also used to examine differences in breathing event indices (AHI, RDI) related to baseline ISI and ISI change scores. Correlation coefficients determined relationships between continuous variables (age, BMI, PSS and ISI scores, objective sleep indices, and subjective sleep metrics). Post hoc analysis compared insomnia severity scores at intake and posttreatment, based on use status for several different drug categories. A *P*-value of 0.05 was considered statistically significant. Data were analyzed with IBM SPSS Statistics, version 23.0 for Windows (IBM Corporation). All continuous variables are expressed as the mean ± SD or mean 95% confidence interval when indicated.

## Results

### Baseline characteristics

The 96 patients were primarily middle aged [(49.29 ± 12.96) years], Caucasian (57.3%) or Hispanic (32.3%), obese [BMI: (33.95 ± 10.63) kg/m^2^], married or living with a partner (52.1%), females (51.0%) with some college or less (61.5%). Average PSS scores, 30.67 ± 8.11, indicated moderate to severe presumptive PTSD (scores ranged from 21 to 50). Baseline insomnia was moderately severe (ISI average 20.47 ± 5.01); all patients indicated daytime impairment due to insomnia (and suffered from the condition for greater than 6 months), and therefore met the criteria for a chronic insomnia disorder. Subjective sleep metrics reported on the intake questionnaire were also indicative of chronic insomnia: SOL (94.72 ± 92.41) min, SE (71.9 ± 18.0) %, and WASO (126.21 ± 113.93) min. Only 17 patients were not using any medications for sleep at intake, 21 were using one agent, and 58 were using two or more (Table 1). All patients were diagnosed with sleep-disordered breathing: 86 OSA (AHI: 30.97 ± 27.90, RDI: 52.82 ± 34.63); 8 UARS (AHI: 1.86 ± 1.59, RDI: 50.26 ± 42.78); and two patients’ breathing indices were not available from their original sleep center.

### Compliance groupings & PAP modes

Of the 96 patients with objective compliance data and subjective outcomes, 70.8% (C-RU, *n =* 68) were compliant and used their devices on (94.44 ± 6.11) % of nights, averaging (6.81 ± 1.27) h/night (Fig. 1). An additional 12.5% (SC-RU, *n =* 12) would have been deemed compliant based on the total group average [(4.42 ± 0.74) h of use on (78.94 ± 14.71) % of nights], but individually SC-RU patients were noncompliant because they were either just below the required 4 h/night average or they fell short of 70% of nights with use > 4 h. In contrast, 16.7% (NC-MU, *n =* 16) of minimal users used only (29.38 ± 25.46) % of nights or just two nights per week, during which they averaged (2.44 ± 1.26) h/night. There were no systematic differences between the three groups in any baseline characteristics, including socio-demographics and subjective sleep indices. There were also no differences in diagnostic AHI or RDI severity between the three groups (Table 1). Moreover, intake ISI scores were not different based on sleep disordered breathing (SDB) severity: mild SDB (*n =* 39), mean ISI 20.51(4.90); moderate SDB (*n =* 25), mean ISI 20.12(5.33); and severe SDB (*n =* 32), mean ISI 20.69(5.04) (*P = 0.*913).

In total, 40.6% (*n =* 39) were using ABPAP, and 59.4% were using ASV (*n =* 57). (Although ASV use is currently contraindicated for certain CHF patients [68], none of our patients suffered from this cardiac problem.) Statistical comparison of PAP mode among compliance groups was prevented due to small ABPAP samples: C-RU (32 ABPAP and 36 ASV); SC-RU (2 ABPAP and 10 ASV); NC-MU (5 ABPAP and 11 ASV). The average time to follow-up was nearly 1 year (11.89 ± 12.22) months, and the data downloads averaged 2.3 months (71.42 ± 75.70) days of data. There was no significant difference in time to follow-up or data download time span among the groups.

### Main insomnia outcomes

Initial analysis of the three groups using repeated measures 3-way ANOVA revealed a significant main effect (*P* < 0.001) for decreases in ISI scores as well as a group by time interaction (*P = 0.*039). Next, we conducted additional tests with repeated measures ANOVA to test for a group × time interaction by comparing compliant as well as subthreshold complaint groups to the noncompliant reference group. However, due to the small sample sizes in the SC-RU and NC-MU groups, this analysis may have been underpowered to detect differences.

**Fig. 2:**
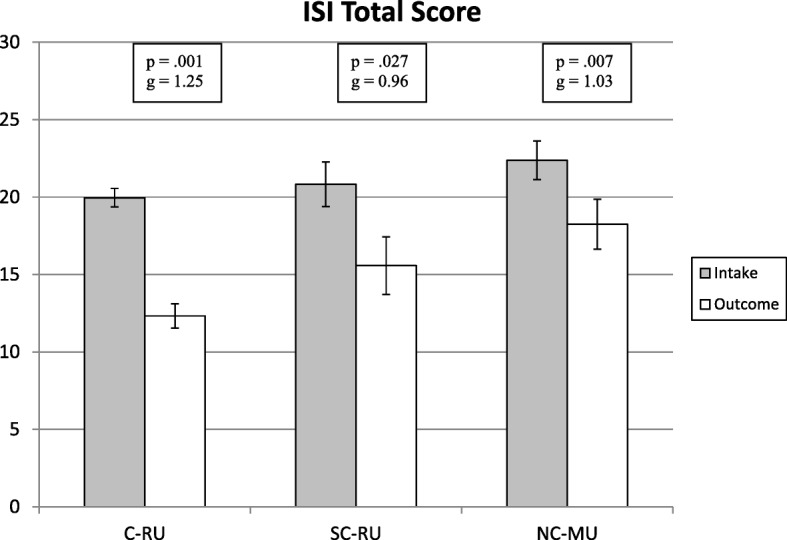
Comparison of mean (standard error) ISI total score, intake vs. Outcome, from compliant regular users (C-RU; *n =* 68), subcompliant regular users (SC-RU; *n =* 12), and non-compliant minimal users (NC-MU; *n =* 16).^a.^ Footnote: ^a^Scores for ISI expressed as the mean (SE) and analyzed with repeated measures ANOVA; *P* and Hedge’s g values for changes in the score from intake to outcome

For the primary outcome within each group, the mean change in insomnia severity significantly decreased on the ISI (C-RU: -7.63, 95% CI 6.30–8.95; *P = 0.*001, g = 1.25; SC-RU: -5.25, 95% CI 1.62–8.88; *P = 0.*027, g = 0.96; NC-MU: -4.13, 95% CI 1.95–6.30; *P = 0.*007, g = 1.03), all with large effects (Fig. 2). However, the only significant difference between the groups occurred in the comparison of the C-RU to the NC-MU group [*F*_1,82_ = 5.762; *P = 0.*019] but not for the SC-RU compared to the NC-MU group [*F*_1,26_ = 0.371; *P = 0.*548] or the C-RU compared to the SC-RU group [*F*_1,78_ = 1.896; *P = 0.*172].

### Residual insomnia outcomes

To examine residual insomnia, we used a strict cutoff of ISI < 8, and overall, only 21.9% of patients were cured of their insomnia: unsurprisingly, 95.2% of these cures were in the C-RU group. Using the more liberal subclinical cutoff of < 12, 39.6% achieved this improvement, of which 84.2% were in the C-RU group. From a clinical standpoint, it is noteworthy that 52.9% of the C-RU group still had clinically relevant residual ISI scores. The thirty-eight patients whose final ISI scores were below the clinical cutoff of 12 averaged significantly (*P = 0.*006) longer nightly PAP use (6.02 ± 3.17) than the 58 patients above the cut-off (4.59 ± 3.09).

To further analyze the possible association between insomnia severity and compliance group status, we explored baseline insomnia measures and identified key subjective metrics that were worse in the NC-MU group than in the C-RU group (Table 1). For example, non-significant medium-sized effects for ISI were noted when comparing the C-RU to the NC-MU (g = 0.47). Continuing this pattern, subjective reports of SE and WASO were also notable with significant moderate to large effects when comparing the C-RU to the NC-MU groups (SE: g = 0.56; WASO: g = 0.89).

Providing further support for an association between sleep breathing treatment and insomnia improvement in the form of a potential dose-response relationship between PAP use and benefits, a correlation showed a significant inverse relationship between weekly hours of PAP use and decreasing insomnia severity (*P = 0.*001, *r* = − 0.321) for the entire sample.

### Posthoc analysis of baseline sleep aids and changes in insomnia severity

Due to the high incidence of sleep aids, prescription and over-the-counter as well as other sedating psychotropic medications, post hoc analysis explored the possible associations between reported intake use and baseline and outcome findings (see Table 1 for the breakdown of categories and incidence). There was no significant difference in baseline ISI scores when comparing use vs. nonuse of any category of medication. In contrast, when comparing change in ISI scores between use and nonuse of each drug category, a trend or significance was noted for smaller improvements among those using hypnotics (*P = 0.*054), seizure medications (*P = 0.*007), and opiates (*P = 0.*061) as sedating agents. No difference existed in baseline ISI scores regarding the number of categories of medications each patient reported; but, when comparing changes in ISI scores, there was a trend for greater improvements (larger decreases in ISI) among patients using no medications than among those who either used one or two categories of medication (− 9.68 ± 5.14 vs. -6.89 ± 5.45, *P = 0.*089 and − 9.68 ± 5.14 vs. -5.59 ± 5.22, *P = 0.*007, respectively). Medication showed no systematic associations with AHI or RDI severity levels.

## Discussion

In this medium-size sample of OSA patients with moderately severe posttraumatic stress symptoms, significant improvements in the severity of insomnia were associated with PAP therapy compliance compared to noncompliance. These observations support a theory that PTSD patients treated for OSA will receive insomnia benefits, though the study design provides only a lower level of evidence. Although underpowered, the small sample of subthreshold compliant patients also showed significant and large effects for insomnia changes. Even the noncompliant group averaging approximately 2 nights per week and 5 h of use per week showed a large effect for change in insomnia severity, albeit this latter group may have simply responded to the attention provided in seeking care or they improved with the passage of time. Ironically, though PAP therapy was associated with decreased insomnia severity, the noncompliant group contained the most severe insomniac patients as measured by the ISI and self-reported SE and WASO, and they appeared to receive the least benefits.

Related to the construct of adherence, the correlation between weekly hours of PAP use and decreases in insomnia severity supports the theory of a potential dose-response relationship [67]. Moreover, the high rate of compliance (70%) among this cohort is noteworthy and indicates a greater potential for PTSD patients to learn to adapt to PAP therapy when provided with the option of advanced PAP therapy. Even using a more conservative calculation based on the original larger sample of 127 patients who filled their PAP prescriptions, the compliance rate still exceeded 50%, a percentage not dissimilar to conventional rates among other cohorts of non-PTSD OSA patients.

Despite the study providing a lower level of evidence due to the retrospective design, this work is consistent with past research demonstrating improvements in posttraumatic sleep disturbance outcomes following PAP therapy use [29, 32, 39, 69–73]. It also adds to the small body of literature on the use of advanced PAP technologies for OSA/UARS in the treatment of psychiatric patients or others susceptible to expiratory pressure intolerance or iatrogenic central apneas [46, 49, 74]. In our setting, sleep technologists address these issues by manually overriding aspects of the autoadjusting algorithms that fail to resolve the dueling problems of residual flow limitation and iatrogenic expiratory pressure intolerance [30, 31, 46, 48, 49, 60]. A nuanced approach requires titrating changes in the 0.2 to 0.4 cm of H_2_O range to independently adjust both inspiratory and expiratory settings [46, 48, 49, 60]. Although our experience reflects case series, we have published on this clinical care model, involving a total of 744 OSA/UARS patients [30, 31, 46, 48, 49] and have treated more than 4000 patients in this manner from 2008 to the present. Clinically, an interesting pearl from our exploration of advanced modes is that ASV appears to eliminate all EPI and all central apneas, whereas ABPAP eliminates nearly all EPI but occasionally shows residual central apneas.

Unfortunately, PTSD symptom follow-up scores were not measured in this clinical work, and therefore no data were available to report on potential changes, but modest reductions in PTSD scores have been described in other research following 6 months of CPAP treatment [73]. Furthermore, it is worth reiterating that insomnia is a major criterion for the diagnosis of PTSD [1], and evidence already exists showing successful insomnia treatment is associated with PTSD improvement [5, 7, 11]. Thus, this physiological therapeutic paradigm may eventually lead to the development of new, sleep-focused treatment pathways for PTSD patients.

Notwithstanding the clinically relevant findings regarding the use of PAP among these insomnia patients, the degree of residual insomnia was pronounced and serves as a clear indication that additional treatment would be needed for this cohort. As many of the patients in the sample were failing a wide assortment of prescription and nonprescription sleep aids, we can imagine how the combination of treatment with PAP and CBT-I would be a very potent regimen [75]. As discussed below, the question emerges on how best to combine these therapies.

It appears that evaluating and treating independent (i.e., comorbid) sleep disorders in PTSD patients is an emerging paradigm with clear-cut opportunities to improve health outcomes in these vulnerable patients [33, 76–78]. However, many questions and some controversies are developing in both the research and clinical realms. The most practical concern is how PTSD patients’ providers including primary care physicians, psychiatrists, and therapists, interact with sleep medicine professionals to expedite access to care at relevant sleep medical centers [45, 79–82]. Other clinically relevant questions refer directly to treatment approaches: How early in the assessment process would a PTSD patient benefit from a sleep specialist evaluation, including testing with polysomnography? Should PTSD patients receive concurrent therapies for the medical disorder, obstructive sleep apnea, while also undergoing psychological therapies for PTSD? Most germane to our findings, when CPAP devices lead to problematic adaptation, how expeditiously should sleep specialists consider advanced PAP therapy modes? Perhaps, most importantly, research already exists on possible sequential treatments, starting with CBT-I for insomnia instead of starting with PAP for OSA, but no studies to our knowledge have explored the concurrent use of PAP and CBT-I in these patients [75]. Rigorous and well-designed research protocols are needed to answer these questions and to determine the clinical effects of sleep disorder therapies on PTSD outcomes. Based on this preliminary research, prospective randomized controlled investigations are warranted to compare the effects of CPAP and advanced PAP modes on PTSD sleep disturbance outcomes as well as on PAP adherence rates. Additionally, in light of the pronounced residual insomnia symptoms noted posttreatment in this cohort, future studies must also include CBT-I arms to better understand the value of sequential vs. concurrent therapies.

The study is limited as a retrospective case series with a nonrandomized control group. Moreover, patients did not undergo formal diagnostic interviews for the determination of a PTSD diagnosis. Randomized control trials must address these limitations; however, due to changing sleep medicine practices, special attention must be given to also compare the home sleep testing/APAP model of care [73] to the in-lab, manual titration of dual-pressure autoadjusting devices [49]. Other limitations include selection bias because some patients were lost to follow-up, data downloads were not available, and posttreatment outcome data were absent in 17% of patients who otherwise met criteria for inclusion in the study. Nevertheless, it is encouraging that 115 patients were deemed users of PAP therapy from a total of 127 patients who filled a prescription for a PAP device (91% use rate). Finally, distinguishing between PAP use and insurance-based adherence criteria requires further research to determine the clinical import.

## Conclusions

Patients with comorbid OSA and moderately severe posttraumatic stress symptoms demonstrated improvements in insomnia severity in association with regular use of advanced PAP therapy. ABPAP and ASV PAP were associated with relatively high use and adherence rates, notwithstanding the retrospective, nonrandomized controlled design. Overall, 9 of 10 patients provided clinical information documenting some degree of PAP use among a total of 127 patients who had filled their PAP prescription. In sum, the main finding of improvement in insomnia severity among posttraumatic stress symptom patients compliant with PAP (and to a smaller degree those at subthreshold compliance) supports the extant literature implicating the potential role of sleep treatment in PTSD patients. However, these findings must be tempered by the obvious need for additional insomnia treatments, most likely CBT-I and possibly pharmacotherapy, in this cohort suffering from comorbid OSA/UARS. Future studies must evaluate whether improvement in sleep outcomes translates into improved PTSD symptoms [33, 83], and studies must discern whether advanced PAP devices lead to greater adherence and better outcomes given their apparent capacity to decrease expiratory pressure intolerance and increase comfort. For vulnerable OSA/UARS patients experiencing CPAP failure, we speculate ABPAP or ASV might need to be considered earlier in the treatment regimen.

## Abbreviations

ABPAP: Auto bi-level PAP
AHI: Apnea hypopnea index
APAP: Autoadjusting CPAP
ASV: Adaptive Servo-ventilation
CBT-I: Cognitive-behavioral therapy for insomnia
CMS: Center for Medicare and Medicaid Services
CPAP: Continuous positive airway pressure
C-RU: Compliant regular PAP users
DME: Durable medical equipment
EPI: Expiratory pressure intolerance
EPR: Expiratory pressure relief
MSAS: Maimonides Sleep Arts & Sciences
NC-MU: Noncompliant minimal PAP users
ODD: Objective data download
OSA: Obstructive sleep apnea
PAP: Positive airway pressure
PSG: Polysomnography
PSS: PTSD symptom scale
PTSD: Posttraumatic stress disorder
RCT: Randomized controlled trial
RDI: Respiratory disturbance index
SC-RU: Sub-compliant regular PAP users
SDB: Sleep disordered breathing
SE: Sleep efficiency
SOL: Sleep onset latency
UARS: Upper airway resistance syndrome
WASO: Wake after sleep onset
